# Prognostic implication of left ventricular hypertrophy regression after antihypertensive therapy in patients with hypertension

**DOI:** 10.3389/fcvm.2022.1082008

**Published:** 2022-12-20

**Authors:** Hyue Mee Kim, In-Chang Hwang, Hong-Mi Choi, Yeonyee Elizabeth Yoon, Goo-Yeong Cho

**Affiliations:** ^1^Division of Cardiology, Department of Internal Medicine, Chung-Ang University Hospital, Chung-Ang University College of Medicine, Seoul, Republic of Korea; ^2^Department of Cardiology, Cardiovascular Center, Seoul National University Bundang Hospital, Seongnam, Gyeonggi, Republic of Korea; ^3^Department of Internal Medicine, Seoul National University College of Medicine, Seoul, Republic of Korea

**Keywords:** left ventricular hypertrophy, hypertension, hypertensive heart disease, left ventricular mass regression, echocardiography

## Abstract

**Background:**

Left ventricular (LV) hypertrophy (LVH) in patients with hypertension is a significant risk factor for cardiovascular mortality and morbidity. However, the prognostic implication of LVH regression after antihypertensive therapy has not been clearly investigated.

**Methods:**

Patients who underwent echocardiography at the time of the diagnosis of hypertension and repeated echocardiography at an interval of 6–18 months were retrospectively identified. LVH was defined as LV mass index (LVMI) >115 g/m^2^ (men) and >95 g/m^2^ (women). LVH regression was defined as LVH at initial echocardiography with normal geometry or concentric LV remodeling at follow-up echocardiography. Cardiovascular mortality, hospitalization for heart failure (HHF), coronary revascularization, stroke, and aortic events were analyzed according to changes in LVMI and geometry.

**Results:**

Of 1,872 patients, 44.7% (*n* = 837) had LVH at the time of diagnosis; among these, 30.7% showed LVH regression. The reduction in LVMI was associated with the reduction in BP, especially in those with LVH at baseline. During follow up (median, 50.4 months; interquartile range, 24.9–103.2 months), 68 patients died of cardiovascular causes, 127 had HHF, and 162 had vascular events (coronary revascularization, stroke, and aortic events). Persistent or newly developed LVH during antihypertensive therapy was a significant predictor of cardiovascular mortality and events, especially HHF. On multivariable analysis, women, diabetes, atrial fibrillation, coronary artery disease, larger LVMI and end-diastolic dimension, and less reduction in systolic BP were associated with persistent or newly developed LVH.

**Conclusion:**

LVH regression in patients with hypertension is associated with a reduction in cardiovascular events and can be used as a prognostic marker.

## Introduction

Hypertensive heart disease (HHD) develops as a result of chronic blood pressure (BP) elevation, which increases the myocardial workload, inducing structural and functional changes, in the absence of other cardiovascular diseases (1). Left ventricular (LV) hypertrophy (LVH) and LV systolic and diastolic dysfunction are the main features of HHD (2, 3). Previous studies indicate that BP reduction with antihypertensive medication can lead to a regression in LVH (1, 2, 4, 5). However, even when a patient’s BP is optimally reduced, LVH regression is not always achieved and *de novo* LVH can sometimes develop (6–8).

LVH is known as an independent prognostic factor of cardiovascular disease (9–11). However, the influence of treatment-induced geometric changes in HHD remains under debate due to conflicting results. Previous cohort studies demonstrated that LVH regression is associated with a decrease in cardiovascular events, suggesting the prognostic value of LVH regression and the reduction in LV mass (12–18). However, several studies reported that LVH regression is only a surrogate marker of BP control, not a prognostic marker (19, 20).

As it remains to be established whether LVH regression is an innocent bystander of HHD or an independent prognostic factor, we aimed to determine the relationship between LVH regression and cardiovascular outcomes in patients with hypertension under antihypertensive treatment.

## Materials and methods

### Study population

Participants comprised consecutive patients diagnosed with hypertension who underwent echocardiography within 1 month of diagnosis and follow-up echocardiography during antihypertensive treatment, at an interval of 6–18 months from the initial echocardiography, at Seoul National University Bundang Hospital and Chung-Ang University Hospital (tertiary care centers in Korea), between 2006 and 2021. Exclusion criteria were as follows: (1) specific cardiomyopathy, such as dilated cardiomyopathy, hypertrophic cardiomyopathy, restrictive cardiomyopathy, ischemic cardiomyopathy, stress-induced cardiomyopathy, Fabry disease, and MELAS syndrome, etc.; (2) significant (≥moderate) valvular heart disease; (3) end-stage renal disease; (4) prior open heart surgery; and (5) any cardiovascular diseases other than HTN that could cause LVH. The use of antihypertensive drugs was defined as the antihypertensive drugs prescribed at both baseline and follow-up echocardiography, with the medication possession ration (MPR) >80% during this period (21). The MPR was estimated by calculating the proportion of pill-days available from filled prescriptions of the antihypertensive during the interval from the date of baseline echocardiography to the date of follow-up echocardiography (21–23).

The study was carried out in accordance with the principles of the Declaration of Helsinki and was approved by the Clinical Research Institute of each hospital (Seoul National University Bundang Hospital: IRB No. B-2206-762-102; Chung-Ang University Hospital: IRB No. 2205-014-19419). The requirement for informed consent was waived because of the retrospective study design.

### Blood pressure measurement and echocardiography

BP was measured using a sphygmomanometer or an oscillometric device, after patients had rested in the sitting position for at least 5 min. BP measurements were recommended twice at 2-min intervals and the mean BP of two measurements were recorded.

Echocardiographic exams were conducted as part of routine clinical care for all patients using commercially available echocardiographic equipment. All patients underwent conventional two-dimensional, M-mode, and color Doppler ultrasonography in accordance with American Society of Echocardiography guidelines (24). LV end-diastolic dimension (LVEDD), LV end-systolic dimension (LVESD), and wall thickness were obtained using M-mode or two-dimensional images. Relative wall thickness (RWT) was calculated as the ratio of twice the posterior wall thickness divided by the LVEDD, and a value over 0.42 was defined as increased RWT. LV mass was estimated using Devereux’s formula, and LV mass was indexed to the body surface area to obtain the LV mass index (LVMI) (25). LV end-diastolic and end-systolic volumes were calculated from apical two-chamber and four-chamber views, and the LV ejection fraction (LVEF) was measured using the Simpson’s biplane method. Left atrial (LA) volume was determined using the biplane area-length method at ventricular end-systole, and the LA volume index was calculated as the LA volume divided by the body surface area. Right ventricular systolic pressure was estimated from the peak velocity of tricuspid regurgitation with right atrial pressure. Reproducibility in measurement of LVMI was evaluated in 30 randomly selected patients using interclass correlation coefficient (ICC). The ICC for intra-observer and inter-observer variability for LVMI was 0.994 (95% CI 0.987–0.997) and 0.990 (0.978–0.995), respectively (Supplementary Figure 1).

The presence of LVH was defined as an LVMI greater than 115 and 95 g/m^2^ in men and women, respectively (24). Four categories of LV geometry were defined as follows: (1) normal, normal RWT and LVMI; (2) concentric remodeling, high RWT and normal LVMI; (3) concentric LVH, high RWT and high LVMI; and (4) eccentric LVH, normal RWT and high LVMI (26). LVH regression was defined as LVH at the initial echocardiography with normal geometry or concentric LV remodeling at follow-up echocardiography.

### Outcomes

The study population was followed up until May 2022. Hospitalization for heart failure (HHF), coronary revascularization, stroke, aortic events, and cardiovascular death were recorded as primary outcomes. HHF was defined as hospitalization for worsening signs or symptoms of heart failure, requiring the administration of intravenous diuretics or vasodilators. Coronary revascularization included percutaneous coronary intervention and/or coronary bypass surgery. Stroke was diagnosed by typical neurological signs and symptoms, assessed by neurologists, and non-invasive brain imaging findings. Aortic events were defined as aortic rupture, surgical or endovascular aortic repair, and dissection. Data on clinical events were obtained from hospital records reported by physicians, telephone contacts, or national death data.

### Statistical analysis

Baseline characteristics are expressed as numbers and percentages for categorical variables, and as the mean ± standard deviation or median [interquartile range] for continuous variables. Continuous variables were compared between groups using the one-way analysis of variance or Kruskal-Wallis test; categorical variables were compared using the Pearson’s chi-squared test. The association between the change in systolic BP (SBP; △SBP) and the change in LVMI (△LVMI) was assessed using multiple linear regression analysis, with adjustment for age, sex, presence of diabetes, dyslipidemia, chronic kidney disease, and coronary artery disease. Event-free survival analyses were performed using the Kaplan-Meier method with log-rank testing and Cox proportional hazard modeling. Univariable Cox proportional-hazard regression analyses were performed to evaluate the predictive values of each variable, and variables found to be significant on univariable analysis were entered into a multi-variable Cox proportional-hazards regression model using the forward selection method. The hazard ratio (HR) and 95% confidence interval (CI) were calculated. To evaluate the predictors of persistent or newly developed LVH, we created three separate logistic regression models [at the time of the initial diagnosis, at follow-up, and their difference (△)]. Considering the multicollinearity of BP measurements, SBP, rather than diastolic BP (DBP), was used as a parameter in the multivariable logistic analysis, given that the relationship between the change in BP (△BP) and △LVMI as assessed by continuous restricted cubic spline curves showed more relevance for SBP than for DBP.

All statistical analyses were performed using SPSS version 22.0 (SPSS Inc., Chicago, IL, USA) and R programming software version 3.6.1 (The R Foundation for Statistical Computing, Vienna, Austria). Statistical significance was set at *p* < 0.05.

## Results

### Baseline characteristics and echocardiographic measurements

A total of 1,872 patients were included in the final analysis. Baseline characteristics of the study population are summarized in Table 1. The mean age was 64.8 ± 13.1 years and 61.6% were men. LV geometry at baseline was classified as normal in 610 patients (32.6%), concentric remodeling in 425 (22.7%), concentric LVH in 445 (23.8%), and eccentric LVH in 392 (20.9%). There were no significant differences in the prevalence of diabetes mellitus, dyslipidemia, and coronary artery disease across the subgroups by the LV geometry at baseline; however, the prevalence of chronic kidney disease was higher in patients with concentric and eccentric LVH than in patients with normal LV geometry or concentric remodeling. At baseline, the overall mean LVEDD, LV end-diastolic volume, and LVEF were 48.7 ± 6.5, 85.8 ± 36.5 mL, and 58.0 ± 11.7%, respectively. The mean LVMI was 88.4 ± 14.4 g/m^2^, 89.1 ± 14.0 g/m^2^, 140.3 ± 34.9 g/m^2^, and 130.7 ± 28.7 g/m^2^, in normal, concentric remodeling, concentric LVH, and eccentric LVH groups, respectively. Among the total study population, 1,230 patients (65.7%) received renin-angiotensin system blockers, 679 (36.3%) received beta-blockers, 850 (45.4%) received dihydropyridine calcium channel blockers, and 318 (17.0%) received thiazide or thiazide-like diuretics.

**TABLE 1:** Baseline characteristics.

	Total (*n* = 1,872)	LV geometry at baseline				*P*-value
		Normal (*n* = 610)	Concentric remodeling (*n* = 425)	Concentric LVH (*n* = 445)	Eccentric LVH (*n* = 392)	
**Clinical factors**						
Age (years)	64.8 ± 13.1	64.3 ± 12.4	65.6 ± 12.3	62.4 ± 15.2	67.4 ± 11.8	<0.001
Male sex	1,196 (61.6%)	423 (68.6%)	318 (73.6%)	264 (54.8%)	191 (46.6%)	<0.001
Diabetes mellitus	544 (29.1%)	165 (27.0%)	129 (30.4%)	138 (31.0%)	112 (28.6%)	0.492
Dyslipidemia	518 (27.7%)	186 (30.5%)	120 (28.2%)	114 (25.6%)	98 (25.0%)	0.323
Chronic kidney disease	121 (6.5%)	28 (4.6%)	17 (4.0%)	48 (10.8%)	28 (7.1%)	<0.001
Coronary artery disease	374 (20.0%)	120 (19.7%)	90 (21.2%)	80 (18.0%)	84 (21.4%)	0.563
Atrial fibrillation	259 (13.9%)	62 (10.2%)	74 (17.4%)	56 (12.7%)	67 (17.1%)	0.002
Stroke	243 (13.0%)	59 (9.7%)	65 (15.3%)	74 (16.6%)	46 (11.7%)	0.012
**Anthropometrics**						
Body mass index (kg/m^2^)	25.2 ± 3.8	25.1 ± 3.6	25.1 ± 4.0	25.5 ± 4.0	25.0 ± 3.8	0.236
Initial systolic BP (mmHg)	153.1 ± 24.5	145.7 ± 19.5	152.6 ± 88.0	165.8 ± 8.9	150.8 ± 23.3	<0.001
Initial diastolic BP (mmHg)	90.1 ± 18.6	85.8 ± 14.7	89.9 ± 15.1	97.5 ± 23.0	88.7 ± 19.1	<0.001
Initial heart rate (bpm)	72.6 ± 20.3	69.2 ± 23.3	74.6 ± 15.7	73.2 ± 21.0	72.6 ± 20.3	<0.001
F/U systolic BP (mmHg)	129.6 ± 16.3	127.3 ± 15.6	129.3 ± 15.6	134.1 ± 8.1	128.3 ± 14.8	<0.001
F/U diastolic BP (mmHg)	82.9 ± 22.8	79.6 ± 16.0	83.9 ± 17.4	86.7 ± 18.7	82.6 ± 36.4	<0.001
**Laboratory tests**						
Hemoglobin (g/dL)	13.4 ± 2.1	13.5 ± 1.9	13.8 ± 2.0	13.5 ± 2.3	12.7 ± 2.1	<0.001
Blood urea nitrogen (mg/dL)	18.0 ± 9.2	16.6 ± 7.0	17.0 ± 7.0	19.4 ± 10.8	19.6 ± 11.6	<0.001
Creatinine (mg/dL)	0.9 (0.7–1.1)	0.9 (0.7–1.0)	0.9 (0.8–1.1)	0.9 (0.8–1.3)	0.9 (0.7–1.2)	<0.001
GFR (mL/min/1.73 m^2^)	78.4 ± 25.9	84.0 ± 24.0	78.7 ± 24.9	73.1 ± 26.5	75.1 ± 27.6	<0.001
Total cholesterol (mg/dL)	170.6 ± 45.0	163.8 ± 44.6	175.8 ± 43.4	179.7 ± 46.2	165.3 ± 43.6	<0.001
**Echocardiographic findings**						
LVEDD (mm)	48.7 ± 6.5	48.7 ± 4.5	43.3 ± 5.1	48.4 ± 5.5	54.7 ± 6.4	<0.001
LVESD (mm)	32.2 ± 7.9	31.3 ± 5.8	27.7 ± 4.8	32.5 ± 7.3	38.3 ± 10.0	<0.001
LVEDV (mL)	85.8 ± 36.5	79.0 ± 23.4	69.7 ± 20.5	90.1 ± 35.5	108.6 ± 52.2	<0.001
LVESV (mL)	39.0 ± 29.6	32.7 ± 17.4	26.8 ± 10.9	41.4 ± 26.6	59.0 ± 46.0	<0.001
LVEF (%)	58.0 ± 11.7	60.6 ± 9.2	61.2 ± 7.7	56.8 ± 11.7	51.8 ± 15.6	<0.001
RWT	0.4 ± 0.1	0.4 ± 0.04	0.5 ± 0.08	0.5 ± 0.1	0.4 ± 0.05	<0.001
E/e’ ratio	12.8 ± 6.6	11.2 ± 4.5	11.3 ± 5.5	14.5 ± 8.1	15.4 ± 7.5	<0.001
LA dimension (mm)	39.7 ± 7.2	37.9 ± 6.2	37.8 ± 6.7	41.2 ± 7.4	42.3 ± 7.3	<0.001
LA volume index	40.1 ± 22.2	34.0 ± 13.8	33.6 ± 14.6	46.6 ± 31.2	49.3 ± 22.2	<0.001
TRV max (m/sec)	2.4 ± 0.5	2.3 ± 0.4	2.3 ± 0.6	2.4 ± 0.5	2.5 ± 0.5	<0.001
PASP (mmHg)	30.2 ± 9.4	29.9 ± 7.9	27.7 ± 7.8	31.0 ± 11.2	32.6 ± 10.3	<0.001
LVMI (g/m^2^)	109.8 ± 33.7	88.4 ± 14.4	89.1 ± 14.0	140.3 ± 34.9	130.7 ± 28.7	<0.001
F/U LVMI (g/m^2^)	112.5 ± 35.0	100.4 ± 28.6	101.5 ± 28.7	123.5 ± 34.2	130.5 ± 39.6	<0.001
△ LVMI (g/m^2^)	2.7 ± 35.4	12.0 ± 26.7	12.4 ± 27.5	−16.8 ± 41.0	−0.2 ± 38.3	<0.001
**Medication**						
ACE inhibitors	166 (8.9%)	57 (9.3%)	38 (8.9%)	37 (8.3%)	34 (8.7%)	0.948
ARB	1,064 (56.8%)	339 (55.6%)	208 (48.9%)	289 (64.9%)	228 (58.2%)	<0.001
RAS blockers	1,230 (65.7%)	396 (64.9%)	246 (57.9%)	326 (73.3%)	262 (66.8%)	<0.001
Beta-blockers	679 (36.3%)	218 (35.7%)	136 (32.0%)	167 (37.5%)	158 (40.3%)	0.090
DHP-CCB	850 (45.4%)	235 (38.5%)	181 (42.6%)	260 (58.4%)	174 (44.4%)	<0.001
NDHP-CCB	132 (7.1%)	47 (7.7%)	35 (8.2%)	29 (6.5%)	21 (5.4%)	0.359
Thiazide	318 (17.0%)	69 (11.3%)	61 (14.4%)	115 (25.8%)	73 (18.6%)	<0.001
MRA	154 (8.2%)	22 (3.6%)	18 (4.2%)	55 (12.4%)	59 (15.1%)	<0.001
Loop diuretics	254 (13.6%)	44 (7.2%)	39 (9.2%)	63 (14.2%)	108 (27.6%)	<0.001
Statin	1,244 (66.5%)	382 (62.6%)	302 (71.1%)	293 (66.0%)	267 (68.1%)	0.035

Values are given as the mean with standard deviation, median with interquartile range, or number (percentage).

BP, blood pressure; F/U, follow-up; LV, left ventricular; LVH, left ventricular hypertrophy; GFR, glomerular filtration rate; ACE, angiotensin converting enzyme; ARB, angiotensin receptor blockers; DHP, dihydropyridine; CCB, calcium channel blockers; NDHP, non-dihydropyridine; MRA, mineralocorticoid antagonists; LVEDD, left ventricular end-diastolic dimension; LVESD, left ventricular end-systolic dimension; LVEDV, left ventricular end-diastolic volume; LVESV, left ventricular end-systolic volume; LVEF, left ventricular ejection fraction; LVMI, left ventricular mass-index; RWT, relative wall thickness; LA, left atrial; TR, tricuspid regurgitation; PASP, pulmonary artery systolic pressure; RAS, renin-angiotensin system.

### Prevalence of LVH regression and its relationship with BP

**FIGURE 1:**
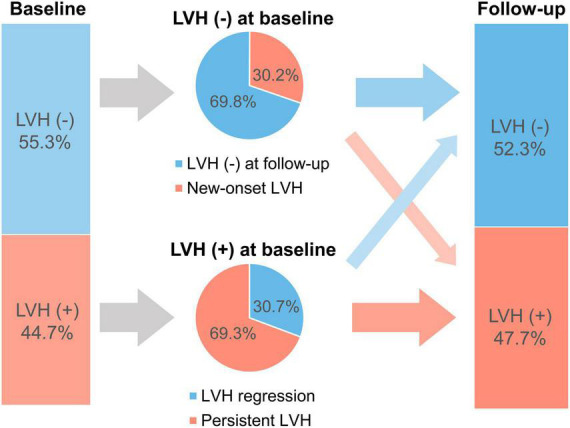
Prevalence of patients with LVH at baseline and follow-up. LVH, left ventricular hypertrophy.

Among patients with concentric or eccentric LVH at baseline (*n* = 837), 30.7% showed LVH regression (Figure 1). Additionally, LVMI was decreased in 64.6% of patients with LVH at baseline. On the other hand, among those without LVH at baseline, 30.2% had newly developed LVH at follow-up echocardiography.

**FIGURE 2:**
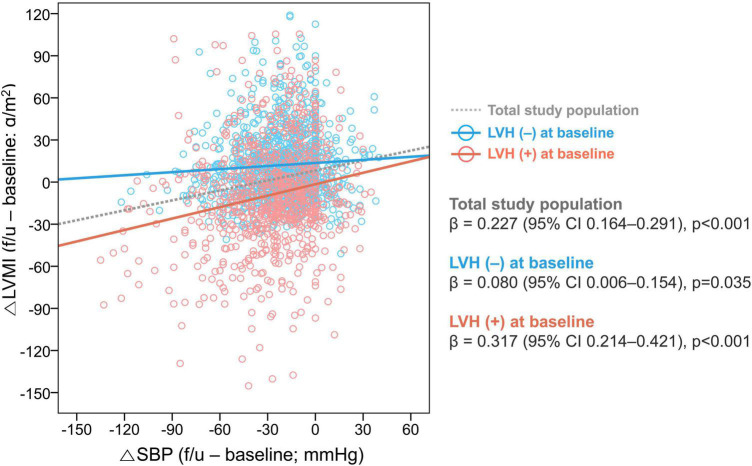
Association between changes in SBP and LVMI. The relationship between the △SBP and △LVMI was assessed in the total study population and subgroups according to the presence of LVH at baseline. LVH, left ventricular hypertrophy; CI, confidence interval; SBP, systolic blood pressure; LVMI, left ventricular mass index.

In the total population, SBP was decreased by −20.0 [−37.0 to −7.3] mmHg and DBP by −6.0 [−19.0 to 4.0] mmHg. Patients with LVH regression had a significantly larger reduction in both SBP and DBP (SBP: −26.0 [−46.5 to −11.0] mmHg; DBP: −10.0 [−25.5 to 1.0] mmHg) compared to that in patients without LVH regression (SBP: −22.0 [−40.0 to −9.0] mmHg; DBP: −6.0 [−20.0 to 7.0] mmHg). The association between △SBP and △LVMI is shown in Figure 2. Patients with a larger reduction in SBP showed a larger reduction in LVMI (β = 0.227, 95% CI 0.164–0.291, *p* < 0.001 by multiple linear regression), and this trend was more robust in patients with LVH at baseline (β = 0.317, 95% CI 0.214–0.421, *p* < 0.001) than in patients without LVH at baseline (β = 0.080, 95% CI 0.006–0.154, *p* = 0.035).

### Baseline and follow-up LV geometry and clinical outcomes

**FIGURE 3:**
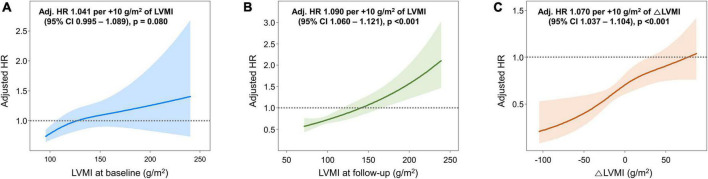
Associations between LVMI and the risk of the composite study outcome. Spline curves showing the adjusted HR for the composite study outcome according to **(A)** LVMI at baseline, **(B)** LVMI at follow-up, and **(C)** △LVMI [(LVMI at follow-up) – (LVMI at baseline)]. HR, hazard ratio; LVMI, left ventricular mass index.

During a median follow up of 50.4 [24.9–103.2] months, 68 cardiac deaths, 127 HHFs, and 162 vascular events (including coronary revascularization, stroke, and aortic events) occurred. Associations between the LVMI and the risk of composite study outcome are shown in Figure 3. The cubic spline curve demonstrated a modest association between the LVMI at baseline and the adjusted HR for the composite study outcome; however, the CI for the HR overlapped 1.0, suggesting a lack of statistical significance (Figure 3A). In contrast, the risk of the study outcome was proportional to the LVMI at follow-up echocardiography and △LVMI across their entire range of values, revealing significant relationships (Figures 3B, C).

**FIGURE 4:**
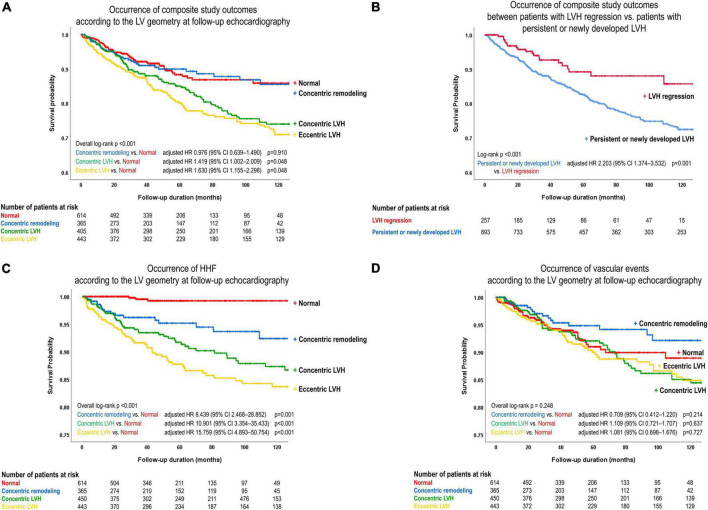
Event-free survival curves. The risk of the composite study outcome was compared **(A)** according to the LV geometry at follow-up echocardiography, and **(B)** between patients with LVH regression at follow-up echocardiography and those with persistent or newly developed LVH. The risk of **(C)** HHF and **(D)** vascular events was compared according to the LV geometry at follow-up echocardiography. LVH, left ventricular hypertrophy; HR, hazard ratio; CI, confidence interval.

Kaplan-Meier survival curves showed similar results when patients were categorized according to LV geometry. Compared to those with normal LV geometry at baseline, patients with concentric and eccentric LVH at baseline, but not those with concentric LV remodeling at baseline, had a higher risk of the composite study outcome (*p* < 0.05) (Supplementary Figure 2). Differences in the prognosis according to LV geometry were also observed when assessed using follow-up echocardiographic data. The risk of the composite study outcome was higher in those with concentric or eccentric LVH at follow-up than in patients with normal LV geometry at follow-up (concentric LVH: adjusted HR 1.419, 95% CI 1.002–2.009, *p* = 0.048; eccentric LVH: adjusted HR 1.630, 95% CI 1.115–2.298, *p* = 0.005) (Figure 4A and Table 2).

**TABLE 2:** Associations between LV geometry and the composite study outcome.

Variables	Multivariable analysis #1			Multivariable analysis #2		
	Adjusted HR	95% CI	*P*-value	Adjusted HR	95% CI	*P*-value
Age (per + 1 year)	1.033	1.023–1.044	<0.001	1.034	1.021–1.046	<0.001
Diabetes mellitus	1.629	1.284–2.065	<0.001	1.923	1.468–2.518	<0.001
Chronic kidney disease	2.185	1.490–3.204	0.001	2.309	1.503–3.547	<0.001
Coronary artery disease	1.393	1.069–1.815	0.014	1.421	1.053–1.916	0.021
Stroke	–	–	–	–	–	–
LVH at baseline	1.306	1.015–1.679	0.038	1.578	1.152–2.162	0.005
**Follow-up LV geometry**						
Normal	*Referenced*					
Concentric remodeling	0.976	0.639–1.490	0.910			
Concentric LVH	1.419	1.002–2.009	0.048			
Eccentric LVH	1.630	1.155–2.298	0.005			
**Changes in LV geometry**						
LVH regression				*Referenced*		
Newly developed or Persistent LVH				2.203	1.374–3.532	0.001

Multivariable analysis #1 was performed using the LV geometry at follow-up echocardiography; multivariable analysis #2 was performed using the changes in LV geometry between baseline and follow-up echocardiography. Multivariable models were adjusted for age, diabetes mellitus, chronic kidney disease, coronary artery disease, stroke, and LVH at baseline.

HR, hazard ratio; CI, confidence interval; LV, left ventricular; LVH, left ventricular hypertrophy.

In order to assess the prognostic value of the change in LV geometry, event-free survival was compared between patients with LVH regression and those with newly developed or persistent LVH. On multivariable analysis, the risk of the composite study outcome was significantly higher in patients with newly developed or persistent LVH than in patients with LVH regression (adjusted HR 2.203, 95% CI 1.374–3.532, *p* = 0.001) (Figure 4B and Table 2; multivariable analysis #2).

### Differential impact of LV geometry on HHF and vascular events

When components of the composite study outcome were assessed separately, the incidence of HHF was higher in patients with abnormal geometry than in those with normal geometry (concentric remodeling: adjusted HR 8.439, 95% CI 2.468–28.852, *p* = 0.001; concentric LVH: adjusted HR 10.901, 95% CI 3.354–35.433, *p* < 0.001; eccentric LVH: adjusted HR 15.759, 95% CI 4.893–50.754, *p* < 0.001) (Figure 4C). However, the incidence of vascular events did not significantly differ among categories of LV geometry (Figure 4D). In addition, compared to patients with LVH regression, those with newly developed or persistent LVH had a significantly higher risk for HHF (adjusted HR 3.129, 95% CI 1.507–6.499, *p* = 0.002) and cardiovascular death (adjusted HR 4.701, 95% CI 1.138–19.421, *p* = 0.032) (Supplementary Figures 3A, B), but no association was found for vascular events (Supplementary Figure 3C).

### Predictors of persistent or newly developed LVH

Given the prognostic value of the change in LV geometry, we assessed the predictors for newly developed or persistent LVH on follow-up echocardiography. As shown in Table 3, female sex, presence of diabetes mellitus, atrial fibrillation, coronary artery disease, LVEDD, and LVMI at baseline were independently associated with newly developed or persistent LVH. Additionally, higher SBP and DBP were associated with persistent LVH on univariable analysis. After adjusting for covariates and eliminating BP variables with multicollinearity, △SBP showed a significant association with persistent or newly developed LVH (adjusted OR 1.065, 95% CI 1.021–1.110, *p* = 0.003 per + 10 mmHg in △SBP). This finding indicates that a larger reduction in SBP (lower △SBP value) is associated with LVH regression, whereas a smaller reduction in SBP is associated with persistent or newly developed LVH.

**TABLE 3:** Predictors of persistent or newly developed LVH.

Variables	Univariable analysis			Multivariable analysis		
	OR	95% CI	*P*-value	Adjusted OR	95% CI	*P*-value
Age (per + 1 year)	1.008	1.000–1.015	0.033			
Male sex	0.426	0.352–0.515	<0.001	0.254	0.202–0.319	<0.001
Body-mass index (per + 1 kg/m^2^)	0.998	0.974–1.022	0.869			
Diabetes mellitus	1.263	1.034–1.542	0.022	1.272	1.013–1.599	0.039
Chronic kidney disease	1.440	0.994–2.087	0.054			
Atrial fibrillation	1.608	1.233–2.097	<0.001	1.662	1.226–2.253	0.001
Coronary artery disease	1.317	1.050–1.653	0.017	1.668	1.286–2.162	<0.001
Baseline LVEDD (per + 1 mm)	1.080	1.063–1.097	<0.001	1.032	1.011–1.054	0.003
Baseline LVMI (per + 1 g/m^2^)	1.028	1.025–1.032	<0.001	1.031	1.026–1.036	<0.001
**Baseline BP**						
Systolic BP (per + 10 mmHg)	1.035	0.998–1.072	0.062			
Diastolic BP (per + 10 mmHg)	1.010	0.963–1.059	0.696			
**Follow-up BP**						
Systolic BP (per + 10 mmHg)	1.092	1.035–1.153	0.001			
Diastolic BP (per + 10 mmHg)	1.046	0.998–1.096	0.060			
**△BP**						
△Systolic BP (per + 10 mmHg)	1.075	1.019–1.134	0.008	1.065	1.021–1.110	0.003
△Diastolic BP (per + 10 mmHg)	1.050	1.008–1.094	0.021			

BP, blood pressure; OR, odds ratio; CI, confidence interval; LVH, left ventricular hypertrophy; LVEDD, left ventricular end-diastolic dimension; LVMI, left ventricular mass-index.

**FIGURE 5:**
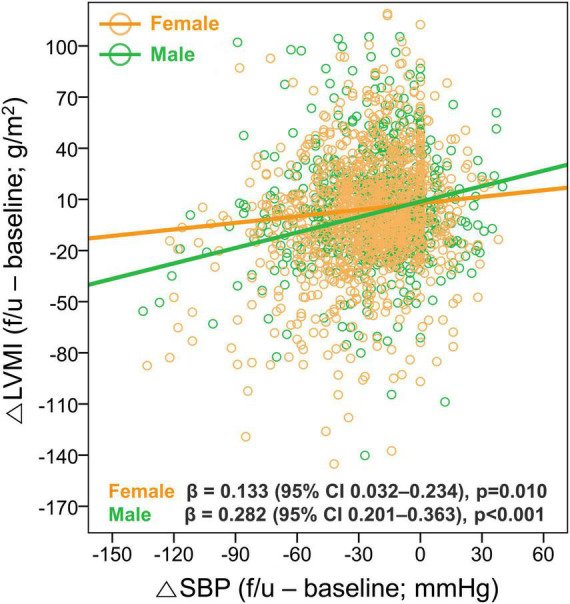
Sex difference in the association between changes in SBP and LVMI. The relationship between the △SBP and △LVMI was assessed according to sex. LVH, left ventricular hypertrophy; CI, confidence interval; SBP, systolic blood pressure; LVMI, left ventricular mass index.

Given the significant association between female sex and the newly developed or persistent LVH, we further assessed the sex differences in the changes in LV geometry. Although there was no sex-difference in initial and follow-up BP, the proportions of LVH at both baseline and follow-up were significantly higher in female patients, resulting in a lower rate of LVH regression. It was also noted that, while the reduction in SBP was associated with a larger reduction in LVMI in both male and female patients, the degree of LVMI reduction (△LVMI) according to the given reduction in SBP (△SBP) was smaller in female than male patients (β = 0.133, 95% CI 0.032–0.234, *p* = 0.010 for female patients; β = 0.282, 95% CI 0.201–0.363, *p* = 0.001 for male patients) (Figure 5). However, the associations between the LVMI and the risk of composite study outcome are similar in both men and women (Supplementary Figure 4).

## Discussion

In the present study, we investigated the associations between changes in BP and LVMI and LV geometry in patients with hypertension, and assessed the prognostic impact of the changes in LV geometry and LVMI. The major findings were as follows: (1) the reduction in BP is proportional to the reduction in LVMI; (2) the reduction in LVMI, or improvement in LV geometry, is associated with a better prognosis, which is mainly derived from the reduced HHF; and (3) the persistence of LVH despite antihypertensive treatment is an independent prognostic factor for adverse outcomes. Our findings highlight the clinical importance of the change in LV geometry among patients with hypertension, not only as an indicator of target organ damage, but also as a relevant surrogate marker of treatment response and independent prognostic marker.

### Development and regression of LVH in patients with hypertension

According to the previous literature, the prevalence of LVH varies 15–45% among patients with hypertension: the prevalence of LVH is lower in population-based study, and higher in patients with untreated or poorly controlled hypertension (27, 28). Due to chronically increased mechanical stress and neurohormonal activation, the LV undergoes structural changes, including an increase in the size of cardiomyocytes, alterations in the extracellular matrix, accumulation of fibrosis, and abnormalities in the intramyocardial coronary vasculature (29). These pathologic changes lead to LV structural remodeling, as well as impairments in systolic and diastolic function (a condition known as HHD), and thus result in a significant risk of heart failure and cardiovascular events (30). As shown in the present study, the LV geometry category reflects the degree of myocardial damage from hypertension, with the worst systolic and diastolic function parameters in patients with eccentric LVH, followed by those with concentric LVH and concentric remodeling. Further, worse LV geometry category was proportionally associated with worse clinical outcomes. In the present study, the prevalence of LVH was relatively high (44.7%), because the patients were enrolled from tertiary care centers, referred from primary or secondary care level hospitals due to severe hypertension. Thus, our study population could represent high-risk patients with severe hypertension, advanced LV remodeling, and higher risk of cardiovascular events.

Because much of the underlying pathophysiology of LVH is due to the increased afterload, the mitigation of pathologic processes by antihypertensive treatment can reverse LVH in HHD. Indeed, antihypertensive medications recommended in clinical guidelines as first-line therapies (angiotensin converting enzyme inhibitors, angiotensin receptor blockers, diuretics, and calcium channel blockers) demonstrate significant BP lowering effects and LV mass regression (2). However, most previous studies focused on the efficacy of antihypertensive medication in reducing BP and improving the prognosis, but did not evaluate the association between BP reduction and LVMI regression. In the present study, we demonstrated that the degree of BP reduction is associated with the amount of reduction in the LVMI, and that this relationship is more prominent in those with LVH at baseline. Our findings confirm that appropriate antihypertensive treatment can regress LVH, highlighting the reversibility of adverse cardiac remodeling through alleviation of the afterload.

In addition to the significant association between the reduction in BP and the regression of LVH, other factors associated with the changes in LV geometry were female sex, presence of diabetes mellitus, atrial fibrillation, coronary artery disease, LVEDD, and LVMI at baseline. These findings are partly in line with previous studies: according to the sub-studies of the Campania Salute Network, older age, female sex, poor BP control, presence of obesity, and higher baseline LVMI are associated with persistent LVH (31, 32). Although we could not find a significant association between BMI and persistent LVH in the present study, the association between female sex and persistent LVH was noteworthy. In particular, the proportions of LVH at both baseline and follow-up were significantly higher in female patients, resulting in a lower rate of LVH regression. These findings suggest that the presence of LVH in female patients requires more attention than in male patients, and more strict therapeutic strategies might be needed in female patients with established LVH.

### Prognostic value of LVH regression

As the presence of LVH indicates myocardial damage from hypertension and antihypertensive therapies can regress LV mass, it can be assumed that LVH regression translates to an improved prognosis. In particular, according to the Losartan Intervention for Endpoint Reduction in Hypertension (LIFE) study, the use of losartan resulted in lower LV mass, and this was associated with lower rates of clinical endpoints (18). Further, a meta-analysis of 3,139 patients from 5 studies reported that LVH regression was associated with a reduction in cardiovascular events (14). However, there is an ongoing debate on whether LVH regression is an independent prognostic marker in patients with hypertension. A meta-analysis of 12,809 participants from 14 clinical trials showed no significant relationship between the change in LVH and cardiovascular events (19). More recently, an ancillary study of the Systolic Blood Pressure Intervention Trial (SPRINT), which investigated LV structural changes using cardiac magnetic resonance (CMR) imaging, reported that there were no significant group differences in LV mass, function, and myocardial T1 between intensive and standard BP control groups, suggesting that mediators other than LV parameters contributed to improved cardiovascular outcomes with intensive BP control (20). Based on these findings, it can be argued that LV mass reduction, or LVH regression, is a surrogate marker of BP control, but is not a prognostic marker.

In the present study, we demonstrated that risk stratification by the LVMI in patients with hypertension is more relevant when using the assessment at follow-up than that at baseline (Figure 3). Further, changes in LV geometry were significant factors of the prognosis: the amount of reduction in LVMI and the regression of LVH were independently associated with a lower risk of cardiovascular events, whereas persistent LVH was associated with a worse prognosis. Among patients with LVH at baseline, one-third showed LVH regression, and two-thirds showed reduced LVMI, at follow-up echocardiography. Thus, although LVH could be regressed with appropriate BP control, there were certain patients for whom the increased LV mass could not be reversed, and these patients had a worse prognosis. Given the independent association between persistent LVH and adverse outcomes, it can be inferred that more attention should be paid to patients with inadequate LVMI reduction despite antihypertensive treatment.

Interestingly, the higher risk of cardiovascular events in patients with persistent LVH was mainly derived from an increased risk of HHF, rather than vascular events. These findings suggest that irreversible myocardial injury from hypertension, or the failure to achieve LVH regression, is the main reason for the higher risk of cardiovascular events, especially for HHF. According to previous studies, irreversible hypertrophied myocardium reflects the presence of irreversible myocardial fibrosis, which impairs LV systolic and diastolic function, and predisposes the development of heart failure (33). Thus, in patients with a larger proportion of irreversible myocardial injury at baseline, the degree of LV mass regression will be smaller and the probability for persistent LVH is higher, which leads to a worse prognosis. Additionally, LVH regression may improve coronary flow and reduce the risk of cardiac arrhythmia (12), which can also contribute to a lower risk of HHF. However, it should be acknowledged that the benefits of antihypertensive therapy in terms of vascular events may require a longer follow-up duration, and LVH regression may have multifactorial effects on the cardiovascular system. Thus, further studies are needed to assess the long-term effect of LVH regression, as well as its pathophysiologic consequences on various cardiovascular outcomes.

### Implication for clinical practice

The potential prognostic value of the change in LV geometry or LVMI has implications for clinical practice, especially regarding the repeated assessment of myocardial structure and function during antihypertensive treatment. Given that LVH reflects structural and functional alterations of the myocardium and indicates a poor prognosis, echocardiographic assessment of LV geometry is considered as a screening for target-organ damage in patients with hypertension (34, 35). However, as indicated in the guidelines, it is not known whether the echocardiography should be repeated once LVH is noted (34). According to the findings of the present study, the change in LV geometry and LVMI during treatment can be relevant markers for treatment response, as well as an indicator for the presence of irreversible myocardial injury. Thus, echocardiographic assessment of changes in LV geometry and LVMI can be a reasonable strategy in the management of patients with hypertension, especially in those with established LVH. Because the risk of cardiovascular events is associated with changes in LV geometry and LVMI, repeated echocardiographic assessment in patients with hypertension can provide additive information on the treatment response and identification of poor responders (i.e., patients with persistent LVH) for more intensive management.

## Limitations

Firstly, because of the retrospective study design, data collection was carried out over a long period of time, follow-up duration was not consistent and the echocardiographic evaluation was performed with different vendors and sonographers. However, we applied uniform criteria for the study population, and also confirmed excellent reproducibility of the echocardiographic measurements (Supplementary Figure 1). Further, we focused on the changes in BP and echocardiographic parameters in patients under antihypertensive treatment and successfully demonstrated associations between BP reduction, LV mass regression, and the risk of cardiovascular events. Thus, we believe that our findings have clinical relevance. Secondly, we could not provide detailed data on antihypertensive medication regimens and BP measurements, such as their variability. We acknowledge that further research is needed on the impact of combination antihypertensive therapy in terms of LV mass regression and its associations with BP variability measurements. Thirdly, we utilized echocardiography for the assessment of LV geometry and LV mass, but did not perform CMR imaging for the assessment of myocardial fibrosis. Future studies using CMR imaging for the quantitative assessment of myocardial fibrosis in a large study population are required.

## Conclusion

Left ventricular hypertrophy regression in patients with hypertension is associated with a reduction in cardiovascular events. LVH regression is a relevant prognostic marker in patients with hypertension, and thus, repeated echocardiographic assessments in these patients can provide risk stratification and guidance for antihypertensive treatment strategies.

## Data availability statement

The original contributions presented in this study are included in the article/Supplementary material, further inquiries can be directed to the corresponding author.

## Ethics statement

The studies involving human participants were reviewed and approved by the Clinical Research Institute of Seoul National University Bundang Hospital and Chung-Ang University Hospital. Written informed consent from the participants’ legal guardian/next of kin was not required to participate in this study in accordance with the national legislation and the institutional requirements.

## Author contributions

HMK and I-CH researched the data and wrote the manuscript. HMK, I-CH, and H-MC provided essential materials and performed analysis. HMK, I-CH, H-MC, YEY, and G-YC read and approved the final manuscript and contributed in revising the manuscript critically for important intellectual content. I-CH revised the manuscript and had primary responsibility for final content. All authors contributed to the article and approved the submitted version.

## Supplementary material

Supplementary Figure 1 Intra-observer and inter-observer reproducibility of LVMI. Scatterplots and Bland-Altman plots are shown for LVMI measurements. Pearson correlation coefficient (r), intraclass correlation coefficient (ICC), and Bland-Altman bias with limits of agreement (LOA) are provided for intraobserver variability **(A,C)** and for interobserver variability **(B,D)**. CI, confidence interval; LVMI, left ventricular mass index; SD, standard deviation.

Supplementary Figure 2 Event-free survival curves. The risk of the composite study outcome was compared according to the LV geometry at baseline echocardiography.

Supplementary Figure 3 Event-free survival curves between patients with LVH regression at follow-up echocardiography and those with persistent or newly developed LVH. **(A)** HHF, **(B)** cardiovascular death, and **(C)** vascular events.

Supplementary Figure 4 Association between LVMI and the risk of the composite study outcome in women and men. Spline curves showing the adjusted HR for the composite study outcome according to **(A,D)** LVMI at baseline, **(B,E)** LVMI at follow-up, and **(C,F)** △LVMI ([LVMI at follow-up] – [LVMI at baseline]). HR, hazard ratio; LVMI, left ventricular mass index.
