# Supplementary figures and images for: Prognostic implication of left ventricular hypertrophy regression after antihypertensive therapy in patients with hypertension
Source: Front Cardiovasc Med. 2022 Dec 20;9:1082008. doi: 10.3389/fcvm.2022.1082008 (PMC9807809; doi:10.3389/fcvm.2022.1082008)

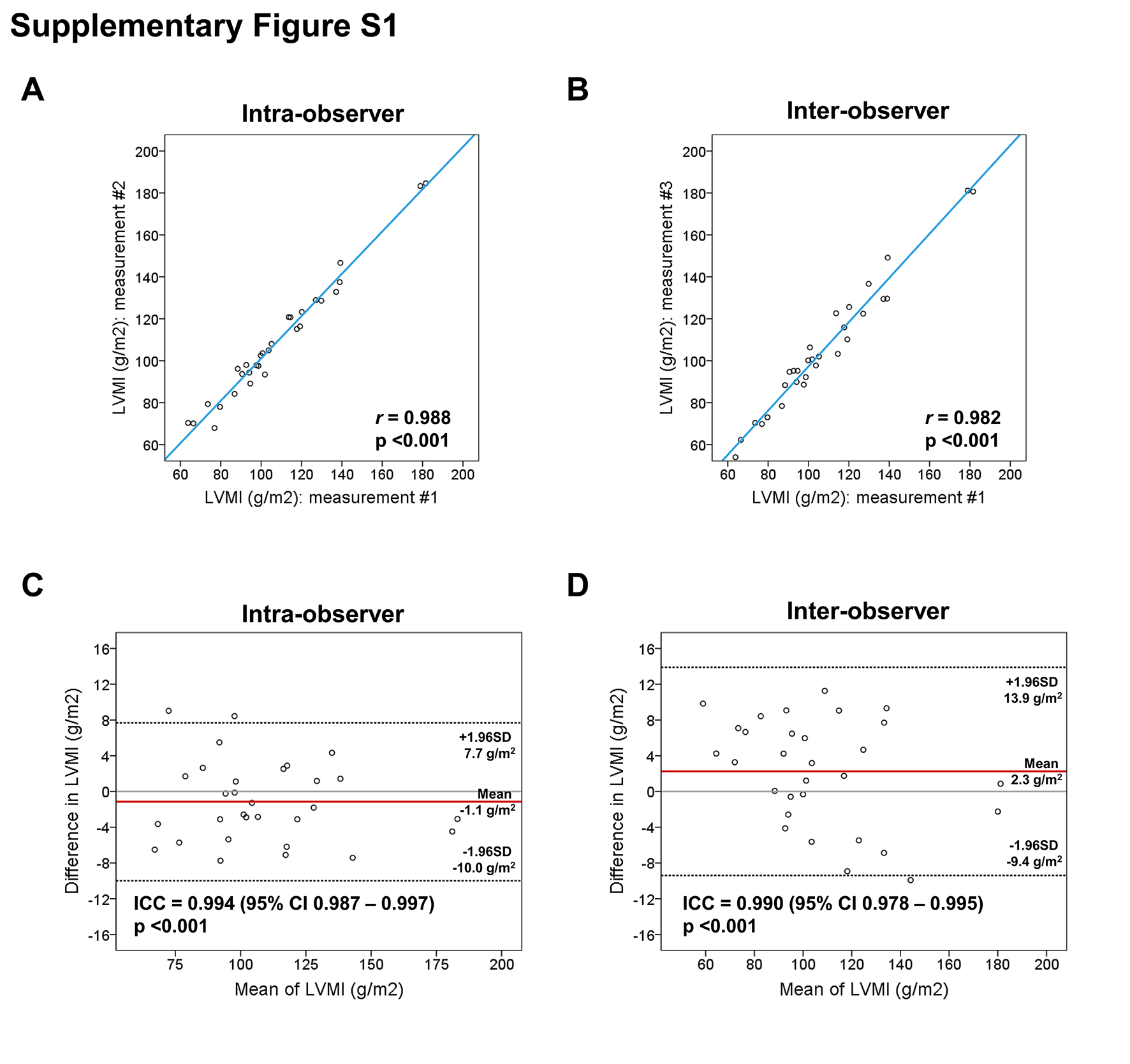

Supplement: Supplementary Figure 1 — Intra-observer and inter-observer reproducibility of LVMI. Scatterplots and Bland-Altman plots are shown for LVMI measurements. Pearson correlation coefficient (r), intraclass correlation coefficient (ICC), and Bland-Altman bias with limits of agreement (LOA) are provided for intraobserver variability (A,C) and for interobserver variability (B,D). CI, confidence interval; LVMI, left ventricular mass index; SD, standard deviation. [file Image_1.TIF]

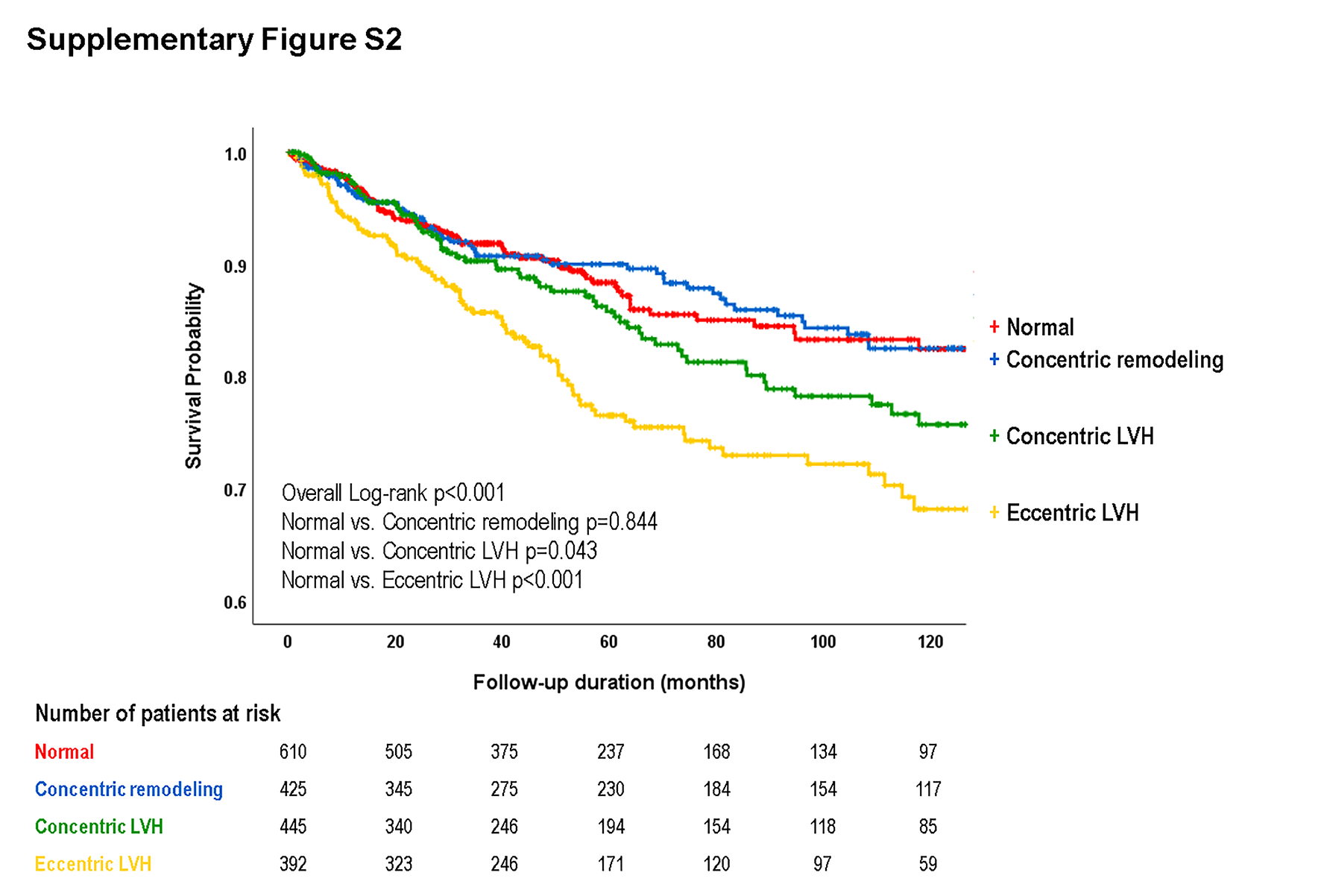

Supplement: Supplementary Figure 2 — Event-free survival curves. The risk of the composite study outcome was compared according to the LV geometry at baseline echocardiography. [file Image_2.TIF]

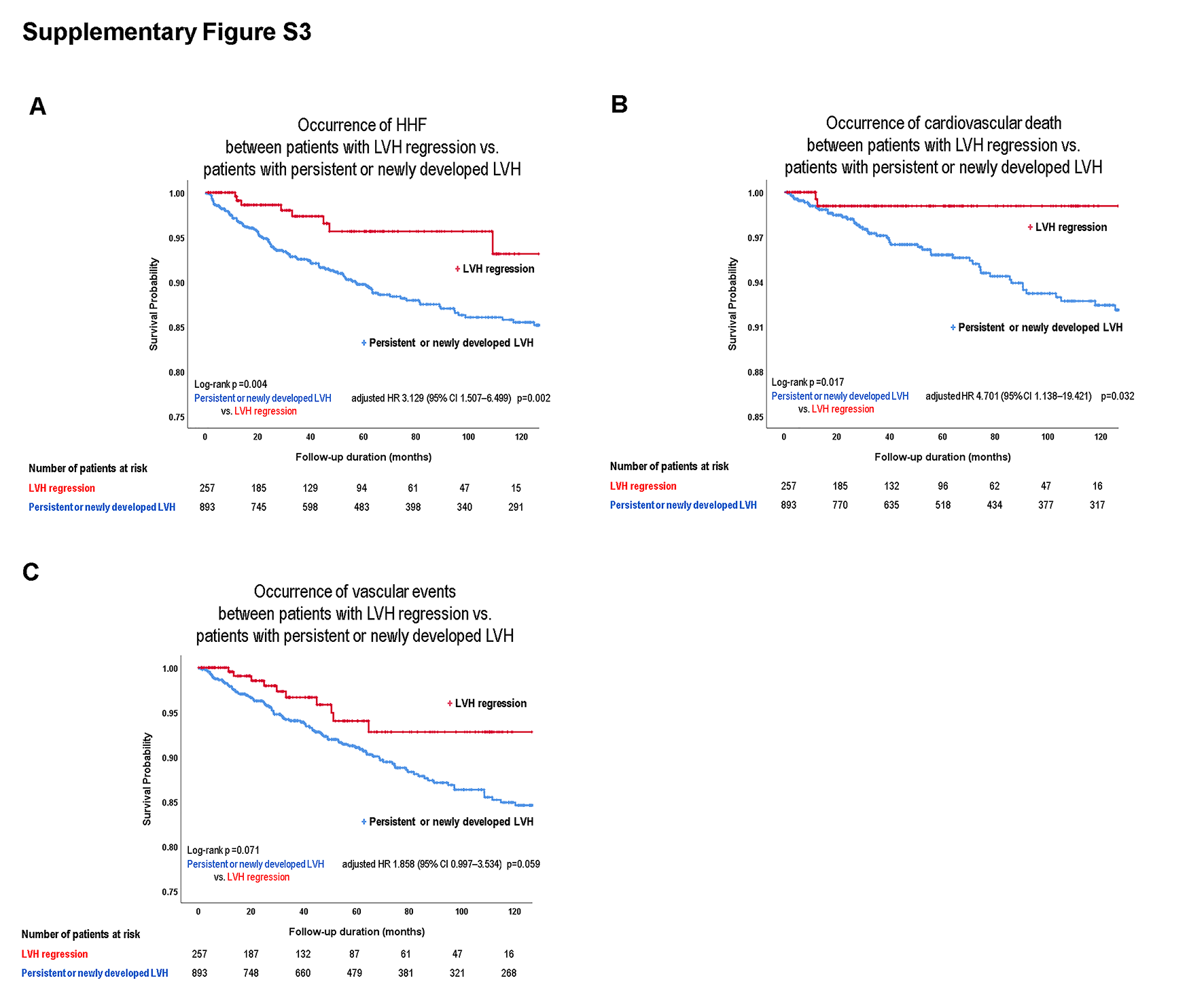

Supplement: Supplementary Figure 3 — Event-free survival curves between patients with LVH regression at follow-up echocardiography and those with persistent or newly developed LVH. (A) HHF, (B) cardiovascular death, and (C) vascular events. [file Image_3.TIF]

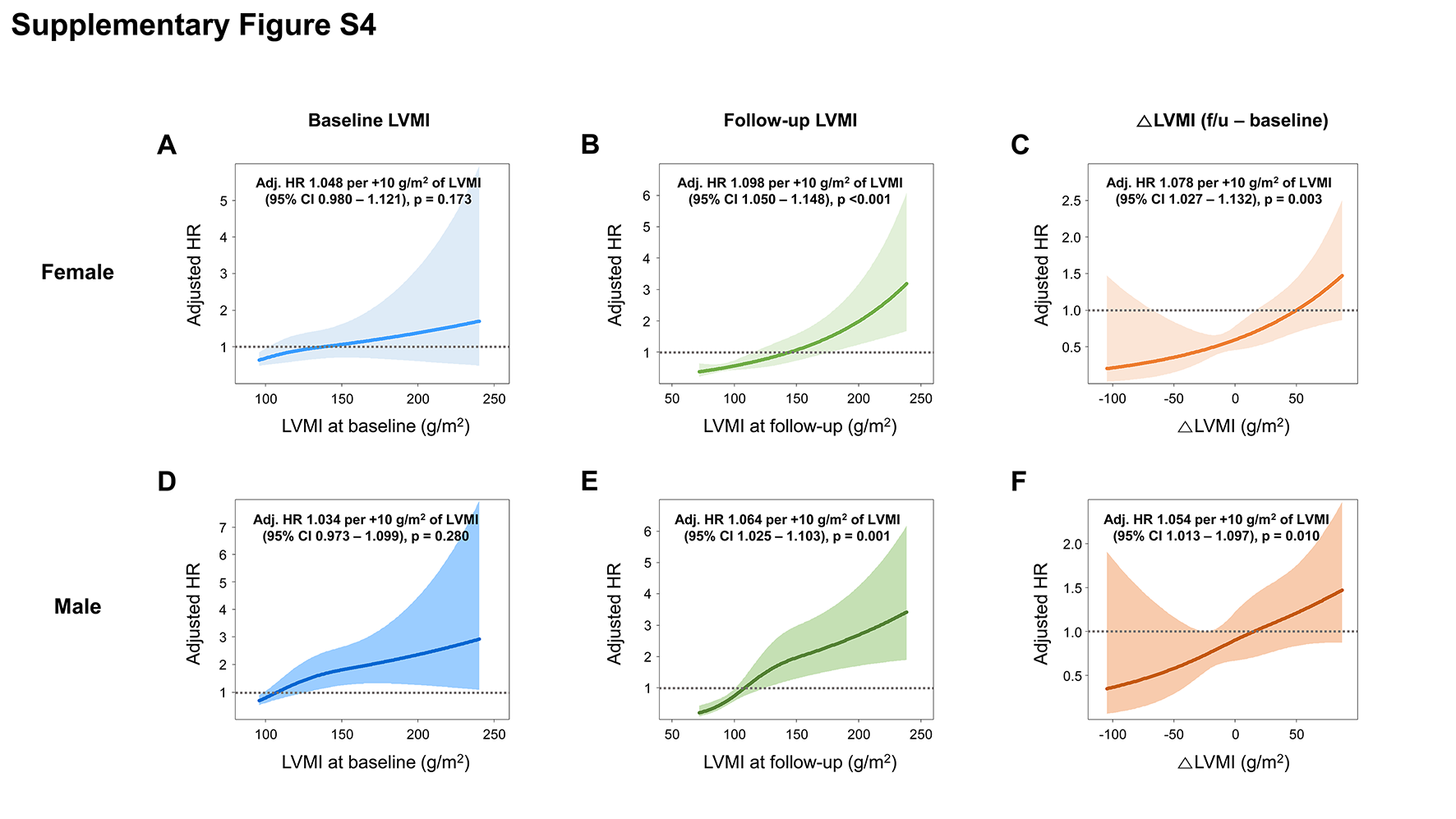

Supplement: Supplementary Figure 4 — Association between LVMI and the risk of the composite study outcome in women and men. Spline curves showing the adjusted HR for the composite study outcome according to (A,D) LVMI at baseline, (B,E) LVMI at follow-up, and (C,F) △LVMI ([LVMI at follow-up] – [LVMI at baseline]). HR, hazard ratio; LVMI, left ventricular mass index. [file Image_4.TIF]
